# On the Effects of InSAR Temporal Decorrelation and Its Implications for Land Cover Classification: The Case of the Ocean-Reclaimed Lands of the Shanghai Megacity

**DOI:** 10.3390/s18092939

**Published:** 2018-09-04

**Authors:** Guanyu Ma, Qing Zhao, Qiang Wang, Min Liu

**Affiliations:** 1 Key Laboratory of Geographical Information Science, Ministry of Education, East China Normal University, Shanghai 200062, China; 51163901076@stu.ecnu.edu.cn (G.M.); 51173901040@stu.ecnu.edu.cn (Q.W.); mliu@geo.ecnu.edu.cn (M.L.); 2 Chongming ECO Institute, East China Normal University, Shanghai 200241, China; 3 School of Geographic Sciences, East China Normal University, Shanghai 200241, China; 4 Laboratory for Environmental Remote Sensing and Data Assimilation, East China Normal University, Shanghai 200062, China; 5 ECNU-CSU Joint Research Institute for New Energy and the Environment, East China Normal University, Shanghai 200062, China

**Keywords:** multi-source remote sensing, coherence, ground deformation

## Abstract

In this work, we focused on the ocean-reclaimed lands of the Shanghai coastal region and we evidenced how, over these areas, the interferometric synthetic aperture radar (InSAR) coherence maps exhibit peculiar behavior. In particular, by analyzing a sequence of Sentinel-1 SAR InSAR coherence maps, we found a significant coherence loss over time in correspondence to the ocean-reclaimed platforms that are substantially different from the coherence loss experienced in naturally-formed regions with the same type of land cover. We have verified whether this is due to the engineering geological conditions or the soil consolidation subsidence in ocean-reclaimed region. In this work, we combine the information coming from InSAR coherence maps and the retrieved temporal decorrelation model with that obtained by using optical Sentinel-2 data, and we performed land cover classification analyses in the zone of the Pudong International Airport. To estimate the accuracy of utilizing InSAR coherence information for land cover classification, in particular, we have analyzed what causes the difference of the InSAR coherence loss with the same type of land cover. The presented results show that the coherence models can be useful to distinguish roads and buildings, thus enhancing the accuracy of land cover classification compared with that allowable by using only Sentinel-2 data. In particular, the accuracy of classification increases from 75% to 86%.

## 1. Introduction

In recent years, global change has become a research hotspot. The development and utilization of land by human beings and the study of land use and land cover (LULC) changes for global environment analyses have been deserved increasing interest. LULC research has great significance in analyzing the effects of human activities on the ecological environment and the feedback of nature [1,2,3].

Remote sensing technology has been widely used in the monitoring of LULC, because of its advantages [4,5,6,7]. Among them, multispectral remote sensing is widely used because of its high spatial resolution and rich spectral information [7,8,9]. However, in some cloudy or rainy weather conditions, it is difficult to obtain optical images. On the other hand, Synthetic Aperture Radar (SAR) technology is valued by all-weather and all-day-time features that can penetrate clouds, smoke, and dust. SAR can obtain surface information over large areas, and it is especially effective in the cloudy and rainy area where traditional optical sensors are difficult to obtain high-quality images for many years. Due to its advantages, SAR images have become very important in remote sensing researches both used alone and in combination with other data [10,11,12,13]. LULC classification is an important area of SAR application. Based on the feature of SAR, lot of works on the application of LULC classification using the SAR data have been done by researchers, and some good results have been achieved [14,15,16,17,18,19,20,21,22].

The study of land cover classification using SAR data can be divided into two classes. The first concerns the use of backscattering intensity information, alone. For instance, Ban et al. (2013) used HJ-1B multispectral optical images and 8 ENVISAT ASAR images, which were acquired in different times and with different side-looking angles, and obtained good classification results by using SVM algorithm [14]. Salehi et al. classified the RADARSAT-2 Polarimetric Radar image by using the SVM algorithm and multi-layer perceptron neural network respectively. The results showed that the accuracy of the classification of the SVM algorithm is better than the multi-layer perceptive neural network [15]. The second class relies on the use of Interferometric Synthetic Aperture (InSAR) technology, using both backscattering intensity and interferometric coherence information. Yocky et al. classified SAR intensity images, and SAR intensity-coherence combined images by using maximum likelihood estimation. They found that adopted coherence-driven method improves the overall classification accuracy of woodland, water, and fields compared with backscattering intensity alone method [16]. Atkinson et al. used ERS1/2 SAR interference data to distinguish the Mackenzie River Delta ecoregion in Canada. They found that the coherence from small to large of the study area was alder, spruce, frozen lake, and road. The temporal decorrelation is the main reason for the decline of coherence [17]. Engdahl et al. studied the land cover classification of Finland’s Nuuksio National Park and Helsinki City by using multi-temporal ERS-1/2 SAR data and concluded that the land cover information provided by the interference coherence map provides richer information than the backscattering intensity [18]. Gray et al. used ESA-1/2 SAR interferometric coherence data to monitor urban changes in Cardiff (South Wales, UK) [19]. Zhou et al. used Sentinel-1A and landsat8 images to extract the winter wheat growing area in southern Jiangsu, China. They found that classification accuracy can be effectively improved by using coherence [20]. In the study of extracting snow cover in the mountainous area, He et al. pointed out that local incidence angle and underlying surface type are closely related to the coherence [21]. To classify different land-cover in Pakistan, Rao Zahid Khalil et al. used Sentinel-1A C-band data comprising of mean backscattering intensity, backscattering intensity change and interferometric coherence obtained from two images, and this resulted classification accuracy increasing up to 87% [22]. These studies demonstrate that coherence is an important factor in improving classification accuracy. At the same time, previous studies have shown that multi-spectral remote sensing does not accurately distinguish roads and buildings when conducting land cover classification. Tuia et al. pointed out that using SVM to classify multispectral remote sensing, the classification accuracy of roads is only 42%, and 49% of roads are misclassified into buildings [23]. Lu et al. pointed out that it is difficult to distinguish between roads and grey roof buildings using hyperspectral and Visible Images [24]. The coherence of roads and buildings is quite different and can be used to improve the classification accuracy of roads and buildings [25].

However, some studies have shown that some factors will affect the decorrelation of the scatterers so that the same land cover has different coherence. As an example, Zebker et al. and Alberga have shown that the random motion of scatterers can lead to loss of coherence [26,27]. Casu et al. demonstrated that severe ground deformation caused by earthquakes could also lead to a loss of coherence [28]. We found this phenomenon on the ocean-reclaimed lands of the Shanghai coastal region. By analyzing a sequence of Sentinel-1 SAR InSAR coherence maps, we found a significant coherence loss over time in correspondence to the ocean-reclaimed platforms that are substantially different from the coherence loss experienced in naturally-formed regions with the same type of land cover. The difference will lead to an error on land cover classification, while InSAR coherence is also used for classification purposes. Previous studies have shown long-term land subsidence occurs in the reclamation area in eastern Shanghai [29,30,31]. We studied whether the continued slow ground deformation, such as land subsidence, also lead to a loss of coherence. And, we studied whether removing the deformation signal can fix the loss of coherence and the errors of land cover classification.

Compared to the coherence information of a single time, the multi-temporal coherence can provide more information for land cover classification and get a temporal decorrelation model. Scientists have done a lot of works. Rocca et al. first proposed a temporal decorrelation model to estimate the possibilities of the interferogram stack technique [32]. Lavalle et al. extended the physical temporal decorrelation model to polarimetric SAR interferometry and successfully used it to extract the structural and dynamic properties of forests [33,34]. In a study of volcanic ash detection, Jung et al. formulated a temporal decorrelation model to differentiate decorrelation sources caused by natural changes [35]. However, there is a lack of similar researches conducted on the temporal decorrelation models that are valid for ocean-reclaimed areas.

Based on above analysis, we can argue there are few studies on whether the land subsidence will affect the coherence of reclamation area, few studies on the temporal decorrelation in reclamation area, also few studies use the temporal decorrelation for reclamation area used in land cover classification. Meanwhile, by analyzing a sequence of Sentinel-1 SAR coherence maps, we found a significant coherence loss over time in the reclaimed platforms that is substantially different from the coherence loss experienced in naturally-formed regions with the same type of land cover. This difference will make the coherence of the same type of land cover have a smaller minimum and a larger span. The same type of land cover has a larger span in coherence, which will reduce the separability of different land cover types. We studied whether land subsidence causes this difference through removing the deformation signal. The relationship between land subsidence, engineering geology and temporal decorrelation were analyzed. Accordingly, a temporal decorrelation model was built taking into account the reclamation area in eastern Shanghai. The temporal decorrelation model improved the accuracy of multi-source remote sensing land cover classification. Shanghai Pudong International Airport was finally selected to do some classification experiments and for the verification of land cover as a validation research.

## 2. Materials and Methods

### 2.1. Study Area and Materials

Shanghai is located at the Yangtze River estuary, surrounded by East China Sea, Hangzhou Bay, Jiangsu and Zhejiang provinces. The city is an important center of economy, transportation, science and technology, industry, finance and trade, and the biggest city in China. Shanghai is rated as one of seven Alpha + cities by Globalization and World Cities Study Group and Network (GaWC). In 2015, Shanghai contributed over 3.68% (about 2.5 trillion) of the GDP and accommodated 1.76% (about 24.15 million) of the population in China [36].

**Figure 1:**
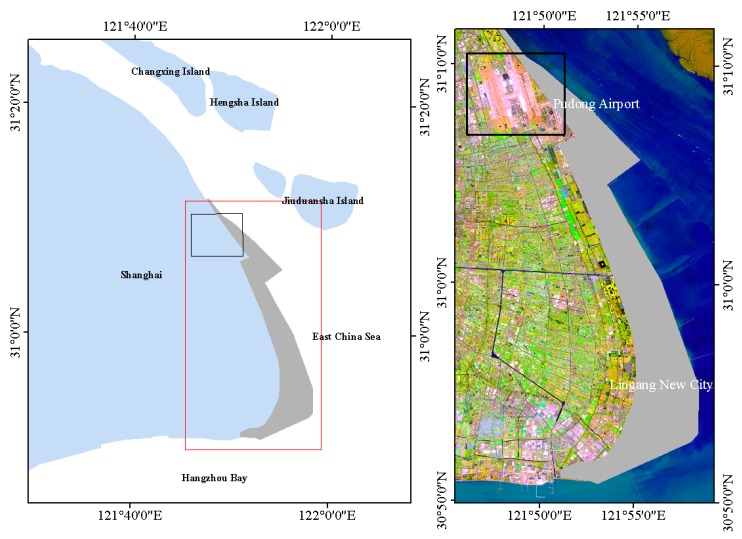
The left image is the location of the study area. The red rectangle is the study area, and the black rectangle is Pudong International Airport. The gray polygon is the area reclaimed from 2002 to the present. The right image is the map of the study area. The background image is a Sentinel2 false color image (the acquisition sate was 28 February 2017; the red channel is the 11th band; the green channel is the 8th band; the blue channel is the 2nd band; the spatial resolution is 10 m × 10 m).

Shanghai has carried out large-scale land reclamation in the past decades. The area of land that has been built is 175 square kilometers, including Pudong International Airport, Nanhui Dongtan, and Lingang New City (Figure 1). The newly formed land area formed by siltation and artificially blow-filling has the characteristics of soil soft, high void ratio, and high moisture content. During the construction of the reclamation project and after the completion of the project, land subsidence will occur due to the self-heavy consolidation.

Pudong International Airport is located in the eastern part of Shanghai. In 2016, the airport handled a throughput of 65.98 million passengers, cargo and mail throughput of 3.43 million tons, movements of 479, 900 take-offs and landing, ranking No. 2, No. 1 and No. 2 respectively in China. The western part of the airport is composed of natural sediments, sandy silt, and silty clay. The eastern part of the airport is an artificial reclamation area filled with loose sandy soil. Under the influence of land reclamation and earth filling properties, the ground deformation will inevitably occur.

In this paper, 32 Sentinel-1A (S1A) images from 20,150,708 to 20,170,826 (C band, orbital direction: Ascending, the angle of incidence: 39°, heading angle: 348°) were used to get coherence data by InSAR and ground deformation data by SBAS algorithm.

### 2.2. Coherence and the Method of Removal of Deformation Signal

When the two SAR images are accurately registered, the conjugate multiplication of the corresponding pixel values can form a (complex) interferogram:

(1) $$\mu_{\mathrm{int}}=\mu_{1}\mu_{2}^{*}=\left|\mu_{1}\mu_{2}\right|\exp(j\Phi )$$

where, *μ*_1_ is the complex image 1, and *μ*_2_ is the complex image 2:

(2) $$\Phi =\Phi_{top}+\Phi_{def}+\Phi_{n}$$

where, Ф*_top_* is the phase related to the topography, Ф*_def_* accounts for the deformation phase, and Ф*_n_* is the phase noise. Note that:

(3) $$\Phi_{top}=\frac{4\pi }{\lambda }\frac{b_{⊥}}{r\sin\theta }$$

(4) $$\Phi_{def}=\frac{4\pi }{\lambda }d_{LOS}$$

where *d_LOS_* is the line-of-sight deformation of the given pixel. When we generate a differential SAR interferogram, we compensate for the topographic phase, and by assuming no orbital phase artifacts are present, the differential phase turns out to be:

(5) $$\Phi =\Phi_{diff}+\Phi_{n}$$

In the case of large deformations, if we calculate the coherence without “compensating the deformation phase” effect the coherence can significantly drop because there are lots of phase fringes in the differential complex interferogram:

(6) $$u_{\mathrm{int}}^{\prime }=u_{1}u_{2}^{*}\exp(-j(\Phi_{top}))=\left|u_{1}u_{2}\right|\exp(j(\Phi_{def}+\Phi_{n}))$$

Causing complete decorrelation in the limit case that the fringe rate is larger than one fringe per pixel [37]. Hence, if we know the value of ground deformation, we may compensate for the deformation phase, by flattening the interferogram doing something like:

(7) $$u_{\mathrm{int}}^{\prime \prime }=u_{1}u_{2}^{*}\exp(-j(\Phi_{def}+\Phi_{n}))=\left|u_{1}u_{2}\right|\exp(j(\Phi_{n}))$$

In this way, the coherence is compensated for by the effects of deformation and only takes into account the other causes of decorrelation noise.

Coherence is the basic measure of the quality of the interferometric data. This is based on the statistical definition of the interferogram, in the window centered at (*i*, *j*) of size 2*n_l_* + 1and 2*n_m_* + 1 respectively, the coherence is defined as follows:

(8) $$c(i,j)=\frac{\left|\sum_{l=-n_{l}}^{n_{l}}\sum_{m=-n_{m}}^{n_{m}}s_{1}(i+l,j+m)s_{2}^{*}(i+l,j+m)\right|}{\sqrt{\sum_{l=-n_{l}}^{n_{l}}\sum_{m=-n_{m}}^{n_{m}}{\left|s_{1}(i+l,j+m)\right|}^{2}•\sum_{l=-n_{l}}^{n_{l}}\sum_{m=-n_{m}}^{n_{m}}{\left|s_{2}(i+l,j+m)\right|}^{2}}}$$

### 2.3. Temporal Decorrelation Model in Ocean-Reclaimed Areas

We can remove the effect of temporal decorrelation by using a temporal decorrelation model, that is a mathematical relationship between the coherence loss and the interferometric temporal baseline (i.e., the time separation of the given InSAR pair).

We experimentally select lots of pixels over ocean-reclaimed platforms, and we do a scatter plot. This allows us to retrieve a mathematical relation between Ctemp and Temporal Baseline:

(9) $$C_{temp}=C_{temp}(t)$$

More specifically, the adopted time-dependent model, m, was originally proposed in [35]. By random-volume-over-ground (RVoG) model proposed by Cloude et al. [38], which is more focused on interpreting the volumetric decorrelation in polarimetric SAR interferometry. The author bound the volumetric decorrelation component described in RVoG model to minimal value so that the observed total coherence is dominated by temporal decorrelation. It is also feasible to using this equation in our study as we want to describe the change of coherence over the temporal baseline.

(10) $$C_{temp}(t)=\frac{1}{1+u}\exp(-\frac{t}{\tau_{v}})+\frac{\mu }{1+\mu }\exp(-\frac{t}{\tau_{g}})$$

where *μ* is the ground-to-volume ratio, *t* is temporal baseline, *τ_v_* is the characteristic time in the volume, and *τ_g_* is the characteristic time in the ground. In our paper, we are using this formula to describe the change of coherence with time. We need to fit 3 parameters *μ*, *τ_v_*, *τ_g_* by coherence and temporal baseline. Levenberg-Marquardt least Squares (LS) minimization technique can solve this problem. In particular, for our experiments, we used the lsqcurvefit code implemented in MATLAB.

## 3. Results and Analysis

### 3.1. Color Characteristics of Different Land Cover on Coherence False Color Images

We used two Sentinel-1A images (their acquisition dates are 27 February 2017 and 11 March 2017, respectively) from the 32 Sentinel-1A images data set. We firstly performed multi-look processing to reduce noise, the spatial resolution of the processed image is 30 m × 30 m. Then, the two images are used to get the coherence. The coherence (1st input) and two intensity images (2nd and 3rd input) were provided as input to output an RGB image. In the output RGB image, the Red channel is the coherence, the Green channel is the backscattering mean intensity, the Blue channel is the backscattering intensity difference (2nd–3rd).

**Figure 2:**
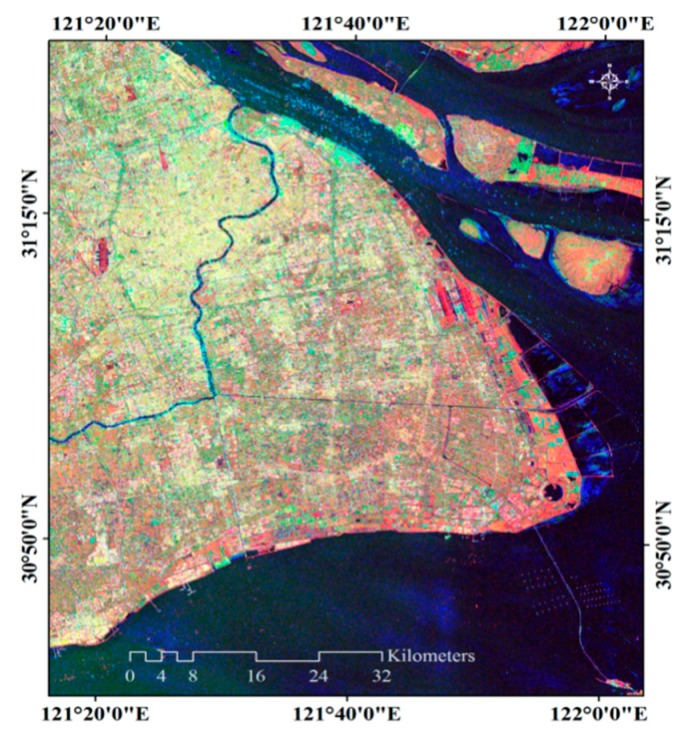
Shanghai coherence false color image (the acquisition dates are 27 February 2017 and 11 March 2017, respectively). The red channel is coherence. The green channel is mean backscattering intensity. The blue channel is backscattering intensity difference. The spatial resolution is 30 m × 30 m).

From the coherence false color image, we can see the following features. Vegetation is usually cyan due to its large backscattering intensity difference. Artificial surfaces mostly appear white due to the high coherence and backscattering intensity. However, it is worth noting that it is obvious in the Figure 2 there are two types of artificial surfaces that present a color that is quite different from most artificial surfaces. One of them is related to the ports. Due to the change of container placed on the port, the coherence is low. Due to the geometric characteristics of the container, the second-order angular reflection that has a strong echo signal is mostly generated, resulting in strong backscattering intensity [39]. This caused a strong backscattering intensity, and the port shows light blue with extremely high brightness. Another is the airport, which is dark red due to low backscattering intensity and low backscattering intensity difference.

We selected samples of three kinds of land cover, port, airport and ordinary artificial surface respectively, and we used Jeffries-Matusita distance (J-M distance) as a parameter to show the separability. We compare the separability of the three kinds of land cover between the coherence false color image and true color optical image. The J-M distance between the ordinary artificial surfaces and the airport is 1.80, the J-M distance between the ordinary artificial surfaces and the port is 1.97, and the J-M distance between the airport and the port is 1.99 in the coherence false color image. In true color images, the J-M distance between ordinary artificial surfaces and the airport is 1.37. The distance between the ordinary artificial surfaces and the port is 0.58, and the J-M distance between the airport and the port is 1.56. The J-M distances in the coherence false color image of the three kinds of land cover are larger than the true color optical images. We can conclude that the coherence false color images are better at extracting ordinary artificial surfaces, ports, and airports.

**Figure 3:**
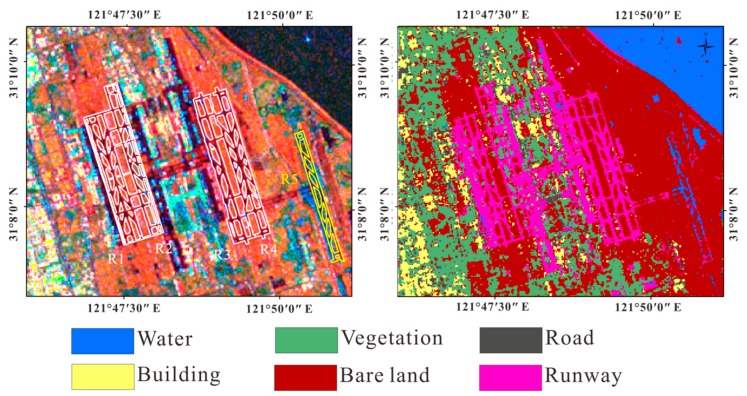
The left image shows the coherence false color image of Pudong International Airport (The acquisition and channel is same as Figure 2. The right image is the classification result of the coherence false color images.

It is noteworthy that we found that at the Pudong International Airport, the R5 runway showed completely different colors from other runways. The R5 runway is located in the reclamation area. We select the Support Vector Machine (SVM) algorithm to classify the coherence false color images. The SVM, first proposed by Vapnik in 1995 [40], is a common identification algorithm. Many previous studies have demonstrated that the SVM algorithm has higher classification accuracy than traditional statistical and neural network algorithms. Salehi et al. found that the classification of the SVM algorithm is better than that of the multi-layer perceptive neural network [14]. Zhang et al. found in the study that the SVM algorithm is superior to Fuzzy C-Means and multi-layer perceptive neural network methods both training speed and image classification accuracy [41]. Based on MATLAB, we wrote SVM code to supervised classification. We selected the RBF kernel function, and get the optimal kernel function parameter width g = 0.5 and the penalty coefficient c = 128 by using grid search method. From the classification results (Figure 3), we can see that the R5 runway was misclassified into the water, which is an unexpected error that should not appear in any research.

In the literature, it is well known that the coherence c can be factorized as the product of different contributions as follows:

(11) $$C=C_{thermal}•C_{temporal}•C_{geom}•C_{doppler}•C_{\mathrm{processing}}$$

where, *C_temporal_* is temporal noise decorrelation, *C_temporal_* is temporal decorrelation, *C_geom_* is geometric decorrelation, *C_doppler_* is Doppler centroid decorrelation, *C_processing_* is data processing decorrelation.

From Equation (11) we can see that coherence is influenced by several factors. The effect of thermal noise on coherence can be determined based on the signal to noise ratio (SNR) of the sensor. Doppler centroid decorrelation can be determined based on Doppler centroid frequency difference and azimuth bandwidth. Data processing decorrelation is mainly caused by registration errors. The decorrelation caused by these three factors affects the entire study area, and there is no difference on the data of thermal noise, Doppler centroid decorrelation, and data processing decorrelation between R5 and other runways.

Geometric decorrelation can be expressed as:

(12) $$C=\{\begin{array}{cc} \frac{B_{⊥,crit}-B_{⊥}}{B_{⊥,crit}} & \left|B_{⊥}\right|\leq B_{⊥,crit}\\ 0 & \left|B_{⊥}\right|≻B_{⊥,crit} \end{array}$$

where, *B*_⊥,*crit*_ is the critical baseline, *B*_⊥_ is the vertical baseline,

(13) $$B_{⊥,crit}=\lambda \frac{B_{R}}{c}r_{1}\tan(\theta -\alpha )$$

where, *α* is the slope angle. From Equations (12) and (13), it can be seen that the slope angle has a greater influence on geometric decorrelation. When classifying by coherence, the influence of geometric decorrelation cannot be ignored. For example, the coherence in a flat region is higher than the undulating region with the same type of land cover. However, the land in the study area is flat, and the slope is unchanged. The geometric decorrelation affects the entire study area, and there is no difference between the R5 and other runways.

Temporal decorrelation is caused by changes in the physical and geometric properties of the scatterers during the acquisition time of two SAR images. Different land cover has different physical properties, which leads to different temporal decorrelation characteristics. Changes in surface roughness over time with different land cover also causes different temporal decorrelation. This is the basis for the use of coherence for land cover classification. However, the factors of temporal decorrelation are complex, and some factors that are not related to land cover may also cause temporal decorrelation in specific and partial regions. For example, severe deformation caused by earthquakes can also cause the coherence of the deformation region to decrease or even be completely decorrelation. This means that the coherence of a type of land cover in the deformation region is much lower than that of the region where no deformation occurs.

The same is true for the Shanghai reclamation area. Previous studies have shown that land subsidence occurs in this area [29,30,31]. If this decorrelation is caused by surface deformation rather than by surface physical properties, it will cause classification errors. Therefore, we studied whether the ground deformation is the main reason for the R5 runway displays different colors, that is, whether this difference can be corrected if we remove the ground deformation signal.

### 3.2. The Influencing Factors of the Difference in the Coherence of the Same Land Cover in the Reclamation Area

**Figure 4:**
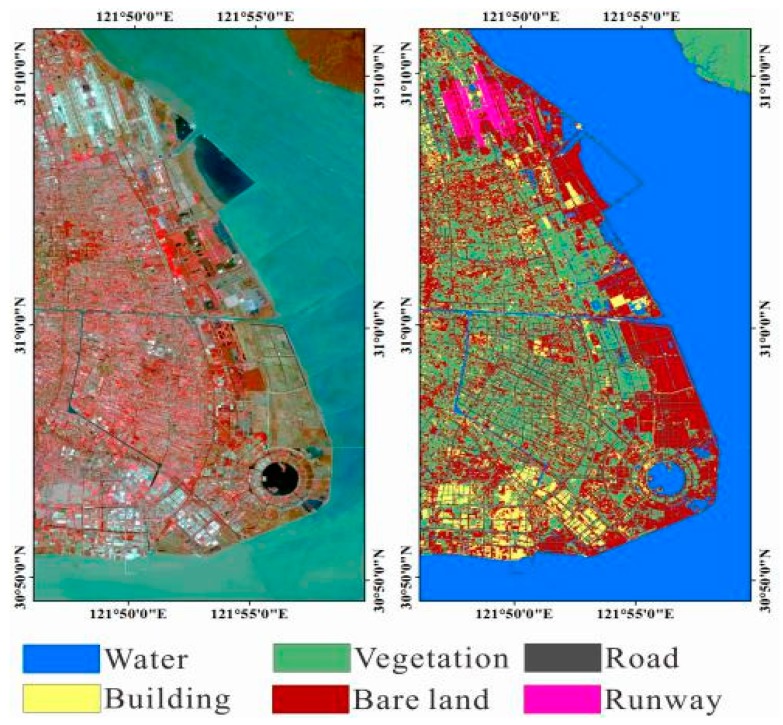
The left image shows the standard false color image of the reclamation area (the acquisition date is 28 February 2017). The red channel is the 8th band. The green channel is the 4th band. The blue channel is the 3rd band. The spatial resolution is 10 m × 10 m. The right image is the map of the land cover type in reclamation area (the spatial resolution is 10 m × 10 m).

**Figure 5:**
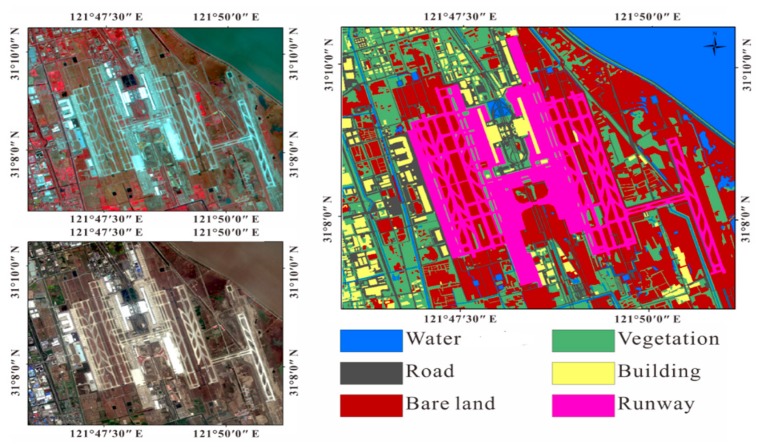
The top left is the standard false color composite image of Pudong International Airport (the acquisition date is 28 February 2017). The red channel is the 8th band. The green channel is the 4th band. The blue channel is the 3rd band. The spatial resolution is 10 m × 10 m. The bottom left is a true color image (the acquisition date is 28 February 2017). The red channel is the 4th band. The green channel is the 3rd band. The blue channel is the 2rd band. The spatial resolution is 10 m × 10 m. The right side is the result of visual interpretation (the spatial resolution is 10 m × 10 m).

We chose Shanghai east reclamation area as a sub-research area (Figure 4). Artificial surfaces are divided into three types: roads, buildings, and airport runways. Non-artificial surfaces are divided into two types according to whether there is vegetation cover or no vegetation cover, and the water. It is divided into six types: water, vegetation, bare soil, roads, buildings and airport runways. We used supervised classification and visual interpretation together to classify the land cover types of the reclamation area to obtain the relationship between the coherence and the temporal baseline of each land cover type. We used the Sentinel-2 standard false color image to classify the reclamation area into five types by SVM. It is notable that they are divided into five types instead of six types. Since multi-spectral images do not distinguish between buildings and roads, buildings and roads are classified into one type, then use the visual interpretation to get the coverage of the road. In Pudong International Airport, based on the true color images of Sentinel-2 images (resolution of 10 m × 10 m) and standard false color images, we visually interpret them and visually interpret the results as verification data of classification results (Figure 5).

#### 3.2.1. The Effect of Removing the Deformation Signal on the Coherence

The reclamation area is located in the eastern part of Shanghai. Under the influence of land reclamation and earth filling, obvious land subsidence will inevitably occur. In this paper, the Small Baseline Subsets (SBAS) [42] technique is used to obtain the land subsidence data of the study area.

**Figure 6:**
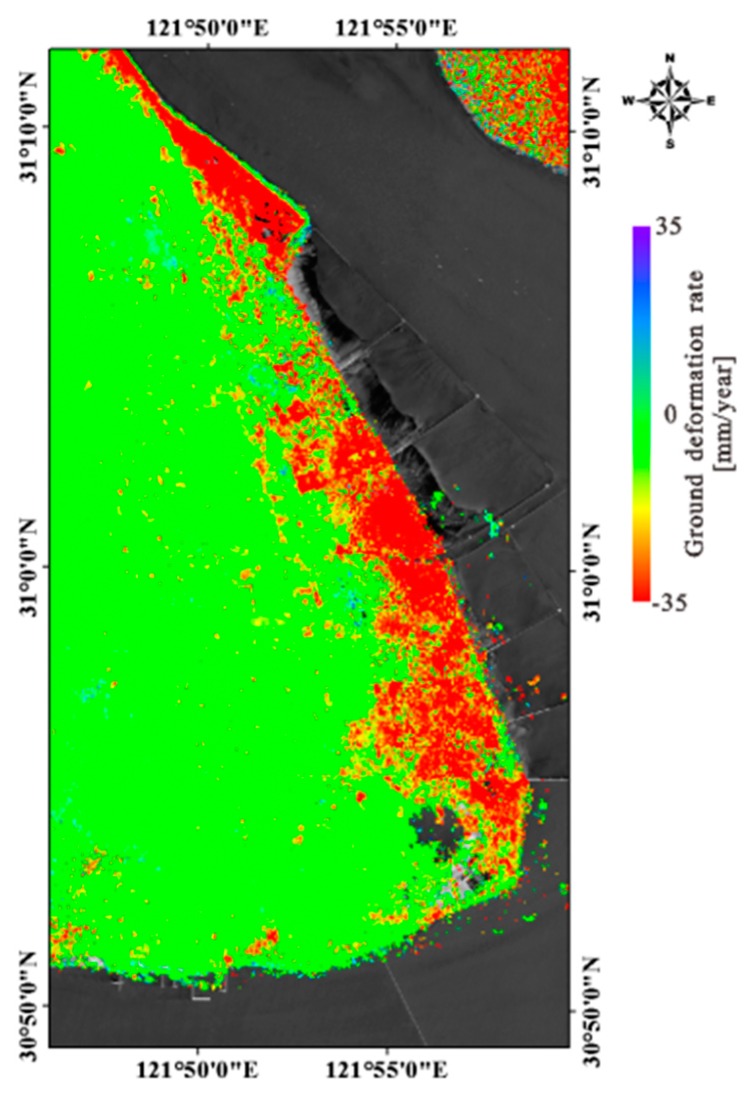
Ground deformation rate in the reclamation area.

We obtained the surface deformation time series using 32 Sentinel-1A (S1A) images from 20,150,708 to 20,170,826 (orbital direction: Ascending, the angle of incidence: 39°, heading angle: 348°). From the results, we can see that land subsidence mainly occurs in the eastern part of the reclamation area. The maximum subsidence rate reaches 35 mm/year (Figure 6).

We consider the area where the vertical ground deformation rate is less than −25 mm/year as land subsidence area, and the area where the vertical ground deformation rate more than −25 mm/year as stable area. We calculated the mean coherence for each land cover type based on visual interpretation results in Pudong International Airport. Mean coherence of land cover types, except water (which is not related at all with land subsidence effects) in the land subsidence area are less than stable area (Table 1). We need to study whether there is a direct relationship between the land subsidence and the decline of the coherence. If the coherence of the same land cover type is equal between the subsidence area and the stable area after the deformation signal is removed, there is a direct relationship between the land subsidence and the decline of the coherence. On the contrary, there is no direct relationship between the land subsidence and the decline of the coherence.

We will use ground deformation information to remove the deformation signal in the interferogram by Equations (1) to (8). Considering that ground deformation is a long-term process, we have selected a group of interference pairs from the 32 Sentinel-1A images data subsets with a longer interval (the acquisition dates are 28 February 2017 and 22 February 2018). We used it to remove the deformation signals. It is worth noting that this process is based on SAR coordinates and that the interferograms and deformation information obtained by SBAS are not multi-looked.

We can see that there is little change on coherence by removing the deformation signal (Table 2). It can be seen that the ground deformation is not the direct cause of the decline in the coherence of the reclamation area, although the coherence of the subsidence area is lower than the stable area.

#### 3.2.2. The Effect of the Engineering Geological Conditions on Coherence

**Figure 7:**
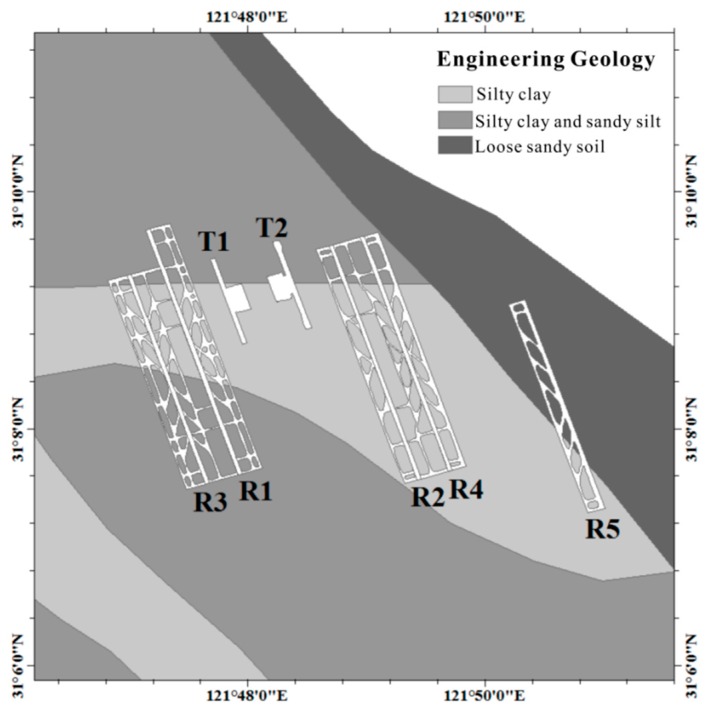
The engineering geology condition of the Pudong International Airport area.

We used the engineering geological map of Pudong International Airport for analysis. The western region of the airport is composed of natural sediments, distributed sandy silt, and silty clay [29,30]. The eastern part of the airport is an artificial foundation, which is filled with loose sandy soil (Figure 7). Comparing with the coherence false color image, we can see that the eastern part of the airport is the area with low coherence coefficient. The engineering geological conditions in the reclamation area are the main factors affecting the time-related coherence. While discussing the time-incoherent model of the reclamation, we should discuss it separately according to different engineering geological conditions.

A previous study by Pepe et al. [30] pointed out that the ground deformation phenomenon of the reclamation area is affected by land reclamation and the filling soil properties. The self-weight consolidation settlement causes the ground deformation in reclamation area. We can find that the newly formed reclamation area is an artificial foundation and filled with loose sandy soil. This will cause both ground deformation and faster temporal decorrelation. This also explains why the same land cover in subsidence area has lower coherence than in stable area, and the coherence will not improve by removing the deformation signal. The use of ground deformation data helps us to approximate distinguish engineering geological conditions in other reclamation areas (without engineering geological maps) to independently discuss the time-incoherent models of reclamation areas.

### 3.3. Temporal Decorrelation Model

Considering that the coherence has obvious time-varying characteristics and richer information of multi-temporal coherence, we used multiple SAR images to obtain the relationship of coherence and temporal baseline. We construct the temporal decorrelation model of temporal baseline and mean coherence of each land cover type. Unless vegetation and bare lands will change with each other over time, the temporal decorrelation model cannot distinguish between vegetation and bare land. We hope that we can effectively distinguish roads and buildings, which are not correctly distinguished by multispectral images. Thus better results can be obtained in multi-source remote sensing classification.

We used the 32 Sentinel-1A data used in the previous SBAS algorithm, and the data acquired at 20,150,708 is the common master image, and the remaining 31 data are processed by the common master image respectively to obtain the coherence.

We have calculated the mean coherence of each type of land cover in the reclamation area (water is excluded because water can be easily distinguished between multi-spectral images and SAR intensities.) according to the previously defined deformation and stable area respectively. Considering some pixels there is serious noise interferes with the whole of the statistical results, we should exclude these pixels. We did the simple linear fitting for coherence time series of each pixel, and only include these pixels that linear fitting error is less than 1. Then we respectively calculated the mean coherence of each land cover type in stable area and reclamation area. Finally we obtained temporal decorrelation model of each land cover type.

**Figure 8:**
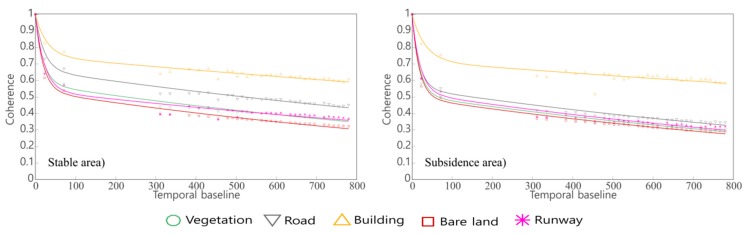
The left is the relationship between temporal baseline and coherence of different land cover types in the stable area. The right is in the subsidence area.

We find that there is a large difference in the temporal decorrelation model of coherence between the deformed reclamation area and the stable western area. We can see that the stable area coherence temporal decorrelation model can better distinguish roads, buildings and other types of land cover. In the deformation area, roads, bare land, airports, vegetation cannot be distinguished, but roads and buildings are still able to be distinguished (Figure 8).

We used Equation (11) to build the temporal decorrelation model. The fitting parameters are shown in Table 3, and the fitting error are shown in Table 4. From Table 3 we can find that the mean *μ* value of the building is 0.36, which is quite different from other land cover types and can be distinguished very well.

### 3.4. Include the Temporal Decorrelation Model into Multi-Source Remote Sensing Image Classification

We obtained a temporal decorrelation model for each pixel and calculated the Euclidean distances with the model of mean coherence of buildings. Using this distance and the model *μ* value as input data to assist multi-source remote sensing image classification. This method can better distinguish between buildings and roads, and reduce the misclassification of roads and buildings.

**Figure 9:**
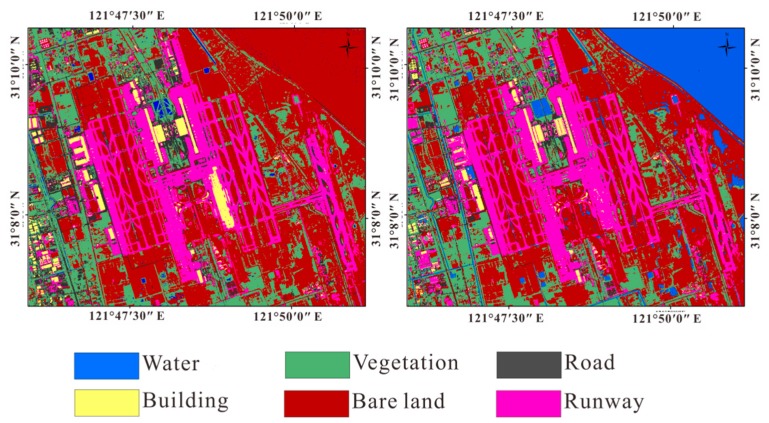
The left image is the classification result of the true color image. The right image is the classification result of the standard false color images.

By way of comparison, we used the same method to classify Sentinel-2 multi-spectral images of true color and standard false-color composite images. Based on MATLAB, we wrote SVM code to supervised classification. We selected the RBF kernel function, and get the optimal kernel function parameter width g = 0.5 and the penalty coefficient c = 128 by using grid search method. The classification results are shown in Figure 9:

Comparing the classification result with the visual interpretation result, the classification accuracy rate of the true color image is 64%, and the KAPPA coefficient is 0.53. The classification accuracy rate of the standard false color images is 75%, and the KAPPA coefficient is 0.68 (Table 5 and Table 6).

From the confusion matrix, we can see that the accuracy of classification for water is only 5%, which cannot identify the water. From the true color images and classification results, we can see that in the East China Sea, which occupies a most portion of water in the upper right part of the image. The true color image shows almost the same brown color as the bare soil due to the sediment concentration.

The accuracy of classification results of standard false color images is better than true color images. Among the classification result of standard false color images, the lowest accuracy is the road, with a correct rate of 38.01%. This is proved again while using multispectral remote sensing to classification, the roads are seriously misclassified as bare.

Compared with the classification results of the true color image, it can be seen that despite the overall accuracy of standard false color image classification results are higher than the true color image classification results, true color image classification accuracy is higher in roads and buildings. It can be seen that different bands and different types of sensors have different advantages for the classification. But both of them cannot distinguish between roads and buildings well. We need to introduce coherence information and temporal decorrelation model into multi-source remote sensing land cover classification.

**Figure 10:**
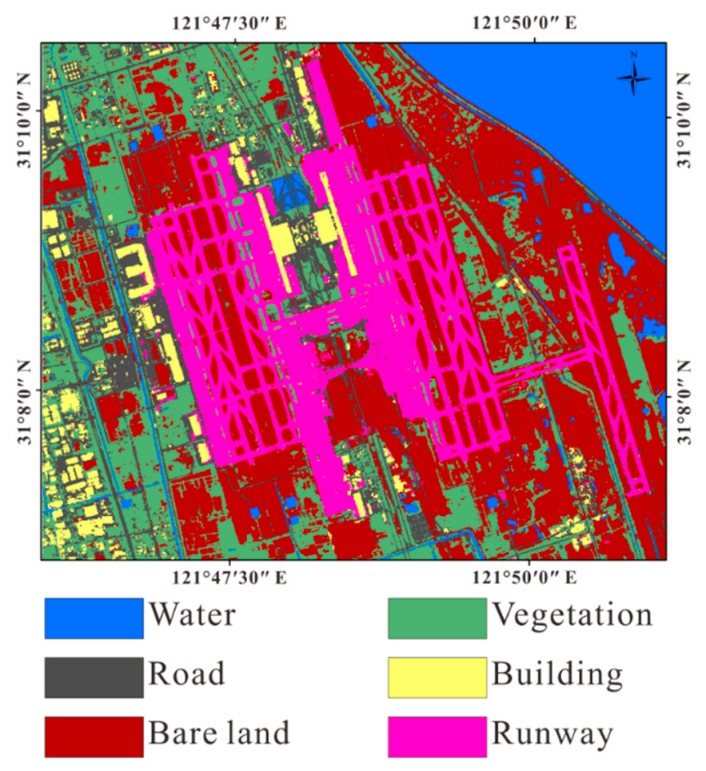
The classification result of Multi-source remote sensing data.

We used the parameters of temporal decorrelation model, coherence data, SAR backscattering intensity data and four bands of Sentinel-2 (red, green, blue and near infrared band) as input data, and used SVM to supervised classification, because SVM has a high popularization ability for high-dimensional input vectors. The classification results is shown in Figure 10.

By comparing the classification result with the visual interpretation result, the classification accuracy rate is 86%, and the KAPPA coefficient is 0.82. Among them, the accuracy rate of the airport is as high as 93%, which can be used to identify the airport well (Table 7). Compared with the classification results of true color images and standard false color images, the overall accuracy rate increased by 22%, 11%, KAPPA coefficient increased by 0.29, 0.14 respectively. 

Multi-source remote sensing fusion can greatly improve the classification accuracy. Using the temporal decorrelation model in multi-source remote sensing land cover classification can greatly improve the recognition rate of buildings and roads.

## 4. Discussion

A significant coherence loss over time in the reclaimed platforms, which is substantially different from the coherence loss experienced in naturally-formed regions with the same type of land cover, was made evident by our investigation. This difference affects the results of the land cover classification. To solve this problem, we analyzed what factors cause this phenomenon. The experimental results have shown that land subsidence, due to ground soil consolidation mechanisms, is not large enough to cause significant decorrelation, but coherence loss is reasonably linked to engineering geological conditions that lead to SAR scenes rapidly-varying over time. However, land subsidence information can be used as an auxiliary proxy to distinguish different engineering geology conditions in the reclamation area. On this basis, we have established a temporal decorrelation model for the subsidence area and the stable area to provide more coherence information. We combined the coherence data and time-incoherent model parameters with the Sentinel-2 data to classify the land cover of Pudong International Airport, which solved the problem that multi-spectral remote sensing could not distinguish roads and buildings accurately. This study can accurately classify land cover in the reclamation area, and can also effectively distinguish roads and buildings.

Compared with the existing research, this paper finds that there is a regional factor affecting the coherence loss in the Shanghai reclamation area, which will lead to the loss of coherence in a specific area. This will affect the land cover classification of the reclamation area if it is directly classified using coherence. In this paper, the temporal decorrelation characteristics of different land cover in reclamation area are analyzed, and the relationship between temporal decorrelation in reclamation area and land subsidence and engineering geological conditions is analyzed. In the end, we effectively improved the classification accuracy by establishing a temporal decorrelation model in reclamation, especially roads and buildings.

It is undeniable that this study has some shortcomings. Firstly, we found that the engineering geological conditions of the reclamation area will lead to regional differences in coherence, but this study did not fix this difference. We only reduce the impact of this difference by establishing a multi-temporal coherence sequence and by discussing separately about the settled and stable regions. Secondly, the results of this study improve the classification accuracy compared with using only Sentinel-2 data, but the accuracy on 86% is not particularly high. This study focuses on discovering new and useful information for land cover classification in reclamation areas, and there is no in-depth study on classification methods, just used the popular SVM method. At the same time, due to the complex land cover of Pudong International Airport, it is difficult to classify accurately. In the study, when visual interpretation is made, individual misjudgments will inevitably occur. Thirdly, the current research is limited to the reclamation area of Shanghai, and this model has not been widely applied to other reclamation areas.

## 5. Conclusions

This work has addressed the intricate relationships between InSAR coherence loss over time and the kind of land-covers in ocean-reclaimed lands. To our knowledge, this study represents one of the first attempts to retrieve a model for the InSAR coherence loss that is specifically intended for the reclaimed lands. Similar experiments have already been performed in other regions on Earth [26,27,28] but in those cases, the most prominent cause of decorrelation was due to the presence of large surface displacement signals, e.g., those due to big earthquakes or volcanic eruptions. The mechanisms that are responsible for decorrelation in ocean-reclaimed areas are completely different and thus deserve an ad-hoc analysis. The implication of InSAR coherence loss for land cover classification analyses has also been addressed. The main outcomes of this study are highlighted in the following.

This study has evidenced that land subsidence is not the main cause of the loss of coherence in the reclamation area, because the spatial extent and the magnitude of the deformation signals in the investigated area are limited. The loss of coherence in the reclamation area can be reasonably caused by the different engineering geology conditions in the reclamation areas and the physical mechanisms at the base of the soil consolidation processes, which are responsible for the observed temporal decorrelation of the investigated scenes. The analysis of sequences of InSAR Sentinel-1 coherence maps over the Shanghai area has demonstrated that some temporal decorrelation models can be retrieved, which can be effectively used for better distinguishing roads and buildings in conventional classification procedures. As a result, we observed how the use of this additional information in multi-source remote land cover classification could effectively improve the classification accuracy of roads and buildings. The results show that the coherence models can be useful to distinguish roads and buildings, thus enhancing the accuracy of land cover classification compared with that allowable by using only Sentinel-2 data. In particular, the accuracy of classification increases from 75% to 86%.

As a future perspective, we plan to quantitatively study the regional differences of coherence caused by engineering geological conditions in the reclamation area. If this can be done, the classification accuracy will be further increased when using coherence for land cover classification in reclamation area. Moreover, we can extend the interference of each SAR image and the master image (the first SAR image) to any two SAR images for interference. This way we will get more coherence information, but this requires more computing time and computing power. In addition, we can extend the study to other bands sensors. In order to verify whether other bands of SAR constellation sensors have the same coherence loss characteristics in reclamation area. We will use the coherence information obtained by the X-band COSMO-SkyMed (CSK) SAR constellation sensors. Finally, we can extend this study to new formed land formed by natural siltation, such as Shekou in Shenzhen and Chongming Dongtan in Shanghai.

## Figures and Tables

**Table 1:** The mean coherence for each land cover type in the stable area and subsidence area.

Land Cover Type	The Mean Coherence	
	Stable Area	Subsidence Area
**Water**	0.226	
**Vegetation**	0.6321	0.553
**Road**	0.591	0.5853
**Building**	0.717	0.6822
**Bare land**	0.633	0.533
**Runway**	0.458	0.357

**Table 2:** The mean coherence for each land cover type in stable area and subsidence area after removing deformation signal.

Land Cover Type	The Mean Coherence	
	Stable Area	Subsidence Area
**Water**	0.226	
**Vegetation**	0.640	0.566
**Road**	0.587	0.587
**Building**	0.712	0.688
**Bare land**	0.641	0.493
**Runway**	0.466	0.394

**Table 3:** The fitting parameters of the mean temporal decorrelation model of different land cover types.

	Land Cover Type	*μ*	*τ_v_*	*τ_g_*
**Stable area**	Vegetation	0.73	1560.92	19.37
	Road	0.51	1844.96	21.78
	Building	0.34	3314.46	28.49
	Bare land	0.86	1402.94	19.44
	Runway	0.85	1912.33	22.09
**Subsidence area**	Vegetation	0.96	1384.96	18.20
	Road	0.81	1506.82	17.86
	Building	0.39	3776.52	32.96
	Bare land	1.01	1334.60	17.79
	Runway	0.90	1385.63	19.99

**Table 4:** The fitting error of the mean temporal decorrelation model of different land cover types.

	Land Cover Type	RMSE	ME
**Stable area**	**Vegetation**	0.014	0.010
	**Road**	0.016	0.011
	**Building**	0.015	0.011
	**Bare land**	0.015	0.011
	**Runway**	0.024	0.016
**Subsidence area**	**Vegetation**	0.015	0.011
	**Road**	0.016	0.011
	**Building**	0.026	0.015
	**Bare land**	0.015	0.010
	**Runway**	0.018	0.013

**Table 5:** Confusion matrix of true color image classification results in Pudong International Airport.

		Classification Result							
		Water	Vegetation	Road	Building	Bare Land	Runway	Entirely	Accuracy
**Visual interpretation**	**Water**	2941	11,073	297	1	50,694	2	65,008	4.52%
	**Vegetation**	919	80,020	6647	232	29,043	367	117,228	68.26%
	**Road**	91	9096	32,141	705	19,044	10,756	71,833	44.74%
	**Building**	24	2363	7703	15,804	2984	4058	32,936	47.98%
	**Bare land**	524	13,290	4569	17	161,063	7519	186,982	86.14%
	**Runway**	0	1706	5073	4133	10,838	75,303	97,053	77.59%
	**Entirely**								64.32%

**Table 6:** Confusion matrix of standard false color image classification results in Pudong International Airport.

		Classification Result							
		Water	Vegetation	Road	Building	Bare Land	Runway	Entirely	Accuracy
**Visual interpretation**	**Water**	59,026	3842	228	1	1910	1	65,008	90.80%
	**Vegetation**	1512	88,716	3846	68	22,644	442	117,228	75.68%
	**Road**	1730	10,552	27,301	494	21,256	10,500	71,833	38.01%
	**Building**	707	3144	5004	14,267	4547	5267	32,936	43.32%
	**Bare land**	2236	13,044	3991	6	160,736	6969	186,982	85.96%
	**Runway**	905	548	4352	716	12,173	78,359	97,053	80.74%
	**Entirely**								75.02%

**Table 7:** Confusion matrix of Multi-source remote sensing data classification results in Pudong International Airport.

		Classification Result							
		Water	Vegetation	Road	Building	Bare Land	Runway	Entirely	Accuracy
**Visual interpretation**	**Water**	59,886	2857	234	35	1983	13	65,008	92.12%
	**Vegetation**	902	92,651	6217	718	15,859	881	117,228	79.03%
	**Road**	411	7821	53,014	1158	7272	2157	71,833	73.80%
	**Building**	72	1958	1889	26,899	1224	894	32,936	81.67%
	**Bare land**	899	11,562	4976	244	167,223	2078	186,982	89.43%
	**Runway**	2	563	2460	349	3342	90,337	97,053	93.08%
	**Entirely**								85.81%
